# Memory Capacity of Networks with Stochastic Binary Synapses

**DOI:** 10.1371/journal.pcbi.1003727

**Published:** 2014-08-07

**Authors:** Alexis M. Dubreuil, Yali Amit, Nicolas Brunel

**Affiliations:** 1 UMR 8118, CNRS, Université Paris Descartes, Paris, France; 2 Departments of Statistics and Neurobiology, University of Chicago, Chicago, Illinois, United States of America; 3 Departments of Statistics and Computer Science, University of Chicago, Chicago, Illinois, United States of America; Hamburg University, Germany

## Abstract

In standard attractor neural network models, specific patterns of activity are stored in the synaptic matrix, so that they become fixed point attractors of the network dynamics. The storage capacity of such networks has been quantified in two ways: the maximal number of patterns that can be stored, and the stored information measured in bits per synapse. In this paper, we compute both quantities in fully connected networks of N binary neurons with binary synapses, storing patterns with coding level 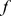, in the large 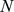 and sparse coding limits (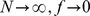). We also derive finite-size corrections that accurately reproduce the results of simulations in networks of tens of thousands of neurons. These methods are applied to three different scenarios: (1) the classic Willshaw model, (2) networks with stochastic learning in which patterns are shown only once (one shot learning), (3) networks with stochastic learning in which patterns are shown multiple times. The storage capacities are optimized over network parameters, which allows us to compare the performance of the different models. We show that finite-size effects strongly reduce the capacity, even for networks of realistic sizes. We discuss the implications of these results for memory storage in the hippocampus and cerebral cortex.

## Author Summary

Two central hypotheses in neuroscience are that long-term memory is sustained by modifications of the connectivity of neural circuits, while short-term memory is sustained by persistent neuronal activity following the presentation of a stimulus. These two hypotheses have been substantiated by several decades of electrophysiological experiments, reporting activity-dependent changes in synaptic connectivity *in vitro*, and stimulus-selective persistent neuronal activity in delayed response tasks in behaving monkeys. They have been implemented in attractor network models, that store specific patterns of activity using Hebbian plasticity rules, which then allow retrieval of these patterns as attractors of the network dynamics. A long-standing question in the field is how many patterns (or equivalently, how much information) can be stored in such networks? Here, we compute the storage capacity of networks of binary neurons and binary synapses. Synapses store information according to a simple stochastic learning process that consists of transitions between synaptic states conditioned on the states of pre- and post-synaptic neurons. We consider this learning process in two limits: a one shot learning scenario, where each pattern is presented only once, and a slow learning scenario, where noisy versions of a set of patterns are presented multiple times, but transition probabilities are small. The two limits are assumed to represent, in a simplified way, learning in the hippocampus and neocortex, respectively. We show that in both cases, the information stored per synapse remains finite in the large  limit, when the coding is sparse. Furthermore, we characterize the strong finite size effects that exist in such networks.

## Introduction

Attractor neural networks have been proposed as long-term memory storage devices [1], [2], [3]. In such networks, a pattern of activity (the set of firing rates of all neurons in the network) is said to be memorized if it is one of the stable states of the network dynamics. Specific patterns of activity become stable states thanks to synaptic plasticity mechanisms, including both long term potentiation and depression of synapses, that create positive feed-back loops through the network connectivity. Attractor states are consistent with the phenomenon of selective persistent activity during delay periods of delayed response tasks, which has been documented in numerous cortical areas in behaving monkeys [4], [5], [6], [7]. A long standing question in the field has been the question of the storage capacity of such networks. Much effort has been devoted to compute the number of attractor states that can be imprinted in the synaptic matrix, in networks of binary neurons [8], [9], [10], [11]. Models storing patterns with a covariance rule [12], [1], [8], [11] were shown to be able to store a number of patterns that scale linearly with the number of synapses per neuron. In the sparse coding limit (in which the average fraction of selective neurons per pattern 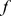 goes to zero in the large 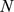 limit), the capacity was shown to diverge as 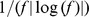. These scalings lead to a network storing on the order of 1 bit per synapse, in the large 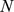 limit, for any value of the coding level. Elizabeth Gardner [10] computed the maximal capacity, in the space of all possible coupling matrices, and demonstrated a similar scaling for capacity and information stored per synapse.

These initial studies, performed on the simplest possible networks (binary neurons, full connectivity, unrestricted synaptic weights) were followed by a second wave of studies that examined the effect of adding more neurobiological realism: random diluted connectivity [9], neurons characterized by analog firing rates [13], learning rules in which new patterns progressively erase the old ones [14], [15]. The above mentioned modifications were shown not to affect the scaling laws described above. One particular modification however was shown to have a drastic effect on capacity. A network with binary synapses and stochastic on-line learning was shown to have a drastically impaired performance, compared to networks with continuous synapses [16], [17]. For finite coding levels, the storage capacity was shown to be on the order of 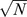, not 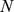 stored patterns, while the information stored per synapse goes to zero in the large 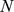 limit. In the sparse coding limit however (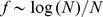), the capacity was shown to scale as 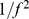, and therefore a similar scaling as the Gardner bound, while the information stored per synapse remains finite in this limit. These scaling laws are similar to the Willshaw model [18], which can be seen as a particular case of the Amit-Fusi [17] rule. The model was then subsequently studied in greater detail by Huang and Amit [19], [20] who computed the storage capacity for finite values of 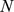, using numerical simulations and several approximations for the distributions of the ‘local fields’ of the neurons. However, computing the precise storage capacity of this model in the large 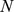 limit remains an open problem.

In this article we focus on a model of binary neurons where binary synapses are potentiated or depressed stochastically depending on the states of pre and post synaptic neurons [17]. We first introduce analytical methods that allow us to compute the storage capacity in the large 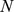 limit, based on a binomial approximation for the synaptic inputs to the neurons. We first illustrate it on the Willshaw model and to recover the well-known result on the capacity of this model [18], [21], [22]. We then move to a stochastic learning rule, in which we study two different scenarios: (i) in which patterns are presented only once - we will refer to this model as the SP (Single Presentation) model [17]; (ii) in which noisy versions of the patterns are presented multiple-times - the MP (Multiple presentations) model [23]. For both models we compute the storage capacity and the information stored per synapse in the large 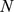 limit, and investigate how they depend on the various parameters of the model. We then study finite size effects, and show that they have a huge effect even in networks of tens of thousands of neurons. Finally we show how capacity in finite size networks can be enhanced by introducing inhibition, as proposed in [19], [20]. In the discussion we summarize our results and discuss the relevance of the SP and MP networks to memory maintenance in the hippocampus and cortex.

## Results

### Storage capacity in the 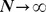 limit

#### The network

We consider a network of 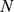 binary 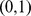 neurons, fully connected through a binary 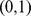 synaptic connectivity matrix. The activity of neuron 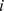 (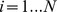) is described by a binary variable, 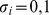. Each neuron can potentially be connected to every other neurons, through a binary connectivity matrix 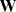. This connectivity matrix depends on 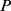 random uncorrelated patterns (‘memories’) 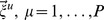 that are presented during the learning phase. The state of neuron 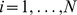 in pattern 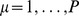 is
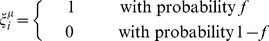
(1)where 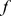 is the coding level of the memories. We study this model in the limit of low coding level, 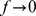 when 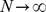. In all the models considered here, 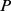 scales as 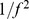 in the sparse coding limit. Thus, we introduce a parameter 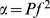 which stays of order 1 in the sparse coding limit.

After the learning phase, we choose one of the 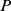 presented patterns 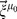, and check whether it is a fixed point of the dynamics:
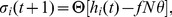
(2)where
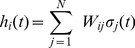
(3)is the total synaptic input (“field”) of neuron 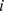, 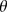 is a scaled activation threshold (constant independent of 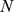), and 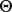 is the Heaviside function.

#### Field averages

When testing the stability of pattern 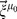 after learning 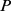 patterns, we need to compute the distribution of the fields on selective neurons (sites 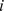 such that 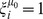), and of the fields on non-selective neurons (sites 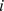 such that 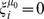). The averages of those fields are 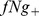 and 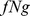 respectively, where
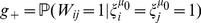
(4)and
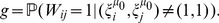
(5)


Pattern 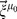 is perfectly imprinted in the synaptic matrix if 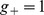 and 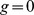. However, because of the storage of other patterns, 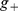 and 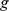 take intermediate values between 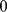 and 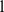. Note that here we implicitly assume that the probability of finding a potentiated synapse between two neurons 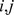 such that 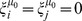 or 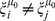 is the same. This is true for the models we consider below. 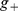 and 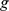 are function of 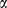, 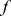, and other parameters characterizing learning.

#### Information stored per synapse

One measure of the storage capability of the network is the information stored per synapse:
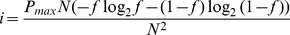
(6)

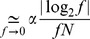
(7)where 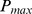 is the size of a set of patterns in which each pattern is a fixed point of the dynamics with probability one. When 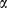 is of order one, for the information per synapse to be of order one in the large 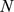 limit, we need to take 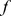 as
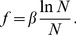
(8)


In this case the information stored per synapse has the simple expression:
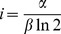
(9)


#### Computing the storage capacity

Our goal here is to compute the size 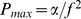 of the largest set of patterns that can be stored in the connectivity matrix. The criterion for storage that we adopt is that if one picks a pattern in this set, then this pattern is a fixed point of the dynamics with probability 1. We thus need to compute the probability 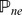 of no error in retrieving a particular pattern 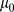. To compute this probability, we first need to estimate the probabilities that a single selective/non-selective neuron is in its right state when the network is initialized in a state corresponding to pattern 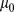. For a pattern with 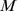 selective neurons, and neglecting correlations between neurons (which is legitimate if 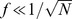 [17]), we have
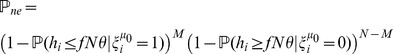
(10)


Clearly, for 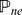 to go to 1 in the large 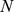 limit, the probabilities for the fields of single neurons to be on the wrong side of the threshold have to vanish in that limit. A first condition for this to happen is 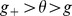 - if these inequalities are satisfied, then the average fields of both selective and non-selective neurons are on the right side of the threshold. When 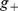 and 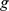 are sufficiently far from 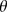, the tail probabilities of the distribution of the fields are
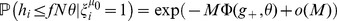
(11)

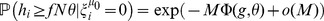
(12)where 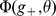, 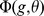 are the rate functions associated with the distributions of the fields (see Methods). Neglecting again correlations between inputs, the distributions of the fields are binomial distributions, and the rate functions are
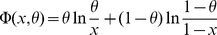
(13)


Inserting Eqs. (11,12,13,8) in Eq. (10), we find that
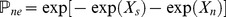
(14)where
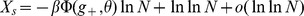


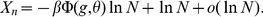
(15)


For 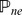 to go to 1 in the large 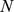 limit, we need both 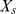 and 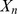 to go to 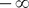 in that limit. This will be satisfied provided
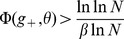
(16)

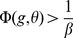
(17)


These inequalities are equivalent in the large 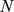 limit to the inequalities
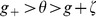
(18)where 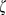 is given by the equation 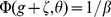.

The maximal information per synapse is obtained by saturating inequalities (16) and (17), and optimizing over the various parameters of the model. In practice, for given values of 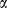, and parameters of the learning process, we compute 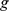 and 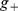; we can then obtain the optimal values of the threshold 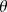 and the rescaled coding level 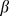 as
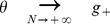
(19)

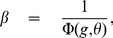
(20)and compute the information per synapse using Eq. (9). We can then find the optimum of 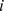 in the space of all parameters.

Before applying these methods to various models, we would like to emphasize two important features of these calculations:

- In Eq. (16), note that the r.h.s. goes to zero extremely slowly as 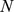 goes to 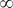 (as 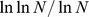) - thus, we expect huge finite size effects. This will be confirmed in section ‘Finite-size networks’ where these finite size effects are studied in detail.
- In the sparse coding limit, a Gaussian approximation of the fields gives a poor approximation of the storage capacity, since the calculation probes the tail of the distribution.

### Willshaw model

The capacity of the Willshaw model has already been studied by a number of authors [18], [21], [22]. Here, we present the application of the analysis described in the previous section to the Willshaw model, for completeness and comparison with the models described in the next sections. In this model, after presenting 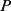 patterns to the network, the synaptic matrix is described as follows: 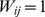 if at least one of the 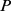 presented patterns had neuron 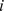 and 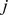 co-activated, 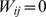 otherwise. Thus, after the learning phase, we have,
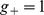


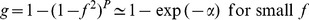
(21)


Saturating the inequalities (19,20) with 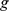 fixed, one obtains the information stored per synapse,
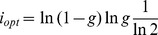
(22)


**Figure 1:**
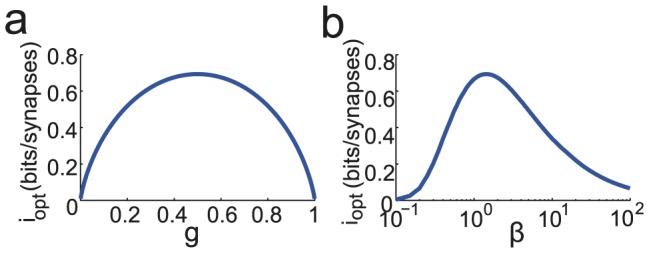
Optimized information capacity of the Willshaw model in the limit 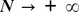. Information is optimized by saturating (19) (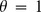) and (20): a. 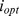 as a function of 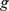, b. 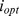 as a function of 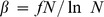.

The information stored per synapse is shown as a function of 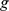 in Figure 1a. A maximum is reached for 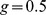 at 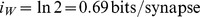, but goes to zero in both the 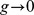 and 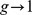 limits. The model has a storage capacity comparable to its maximal value, 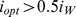 in a large range of values of 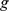 (between 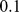 and 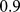). We can also optimize capacity for a given value of 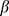, as shown in Figure 1b. It reaches its maximum at 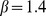, and goes to zero in the small and large 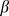 limits. Again, the model has a large storage capacity for a broad range of 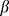, 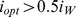 for 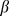 between 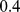 and 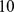.

Previous studies [18], [21] have found an optimal capacity of 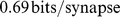. Those studies focused on a feed-forward network with a single output neuron, with no fluctuations in the number of selective neurons per pattern, and required that the number of errors on silent outputs is of the same order as the number of selective outputs in the whole set of patterns. In the calculations presented here, we have used a different criteria, namely that a given pattern (not all patterns) is exactly a fixed point of the dynamics of the network with a probability that goes to one in the large 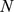 limit. Another possible definition would be to require that **all** the 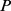 patterns are exact fixed points with probability one. In this case, for patterns with fixed numbers of selective neurons, the capacity drops by a factor of 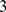, 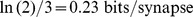, as already computed by Knoblauch et al [22].

### Amit-Fusi model

A drawback of the Willshaw learning rule is that it only allows for synaptic potentiation. Thus, if patterns are continuously presented to the network, all synapses will eventually be potentiated and no memories can be retrieved. In [17] Amit and Fusi introduced a new learning rule that maintains the simplicity of the Willshaw model, but allows for continuous on-line learning. The proposed learning rule includes synaptic depression. At each learning time step 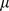, a new pattern 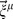 with coding level 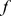 is presented to the network, and synapses are updated stochastically:

- for synapses such that 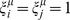:

if 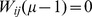, then 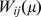 is potentiated to 1 with probability 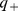; and if 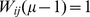 it stays at 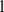.

- for synapses such that 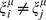:

if 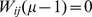, then 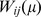 stays at 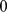; and if 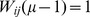 it is depressed to 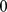 with probability 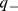.

- for synapses such that 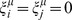, 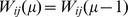.

The evolution of a synapse 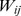 during learning can be described by the following Markov process:
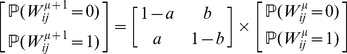
(23)where 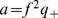 is the probability that a silent synapse is potentiated upon the presentation of pattern 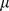 and 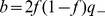 is the probability that a potentiated synapse is depressed. After a sufficient number of patterns has been presented the distribution of synaptic weights in the network reaches a stationary state. We study the network in this stationary regime.

For the information capacity to be of order 1, the coding level has to scale as 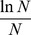, as in the Willshaw model, and the effects of potentiation and depression have to be of the same order [17]. Thus we define the *depression-potentiation ratio* 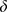 as,
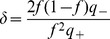
(24)


We can again use Eq. (9) and the saturated inequalities (19,20) to compute the maximal information capacity in the limit 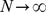. This requires computing 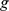 and 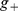, defined in the previous section, as a function of the different parameters characterizing the network. We track a pattern 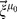 that has been presented 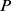 time steps in the past. In the following we refer to 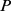 as the age of the pattern. In the sparse coding limit, 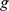 corresponds to the probability that a synapse is potentiated. It is determined by the depression-potentiation ratio 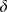,
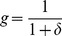
(25)and
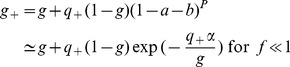
(26)where 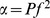. Our goal is to determine the age 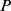 of the oldest pattern that is still a fixed point of the network dynamics, with probability one. Note that in this network, contrary to the Willshaw model in which all patterns are equivalent, here younger patterns, of age 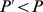, are more strongly imprinted in the synaptic matrix, 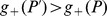, and thus also stored with probability one.

Choosing an activation threshold and a coding level that saturate inequalities (19) and (20), information capacity can be expressed as:
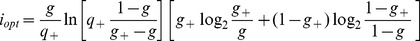


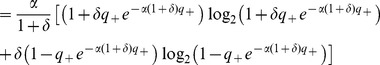
(27)


The optimal information 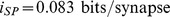 is reached for 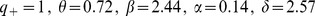 which gives 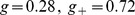.

**Figure 2:**
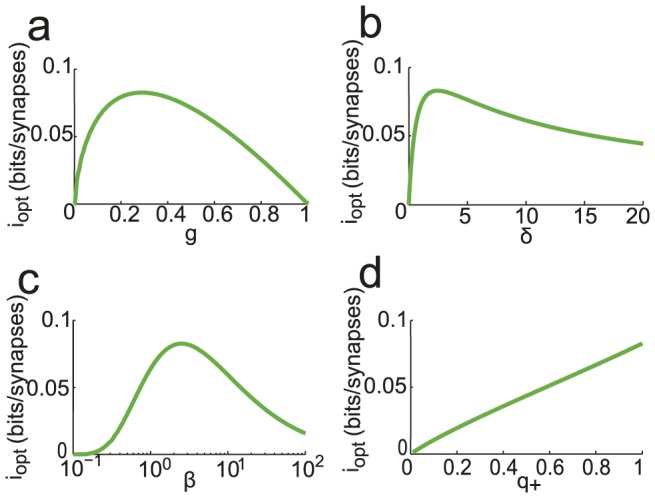
Optimized information capacity for the SP model in the limit 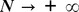. a. 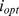 as a function of 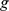, b. 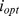 as a function of 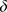, the ratio between the number of depressing events and potentiating events at pattern presentation, c. 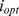 as a function of 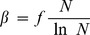, d. 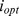 as a function of the LTP transition probability 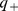.

The dependence of 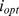 on the different parameters is shown in Figure 2. Panel *a* shows the dependence on 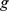 the fraction of activated synapses in the asymptotic learning regime. Panels *b*, *c* and *d* show the dependence on 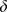, 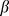 and 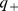. Note from panel *c* that there is a broad range of values of 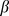 that give information capacities similar to the optimal one. One can also observe that the optimal information capacity is about 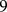 times lower in the SP model than in the Willshaw model. This is the price one pays to have a network that is able to continuously learn new patterns. However, it should be noted that at maximal capacity, in the Willshaw model, every pattern has a vanishing basin of attraction while in the SP model, only the oldest stable patterns have vanishing basins of attraction. This feature is not captured by our measure of storage capacity.

### Multiple presentations of patterns, slow learning regime

In the SP model, patterns are presented only once. Brunel et al [23] studied the same network of binary neurons with stochastic binary synapses but in a different learning context, where patterns are presented multiple times. More precisely, at each learning time step 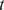, a noisy version 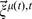 of one of the 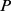 prototypes 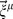 is presented to the network,
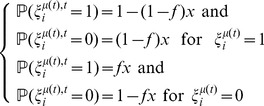
(28)


Here 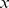 is a noise level: if 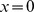, presented patterns are identical to the prototypes, while if 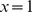, the presented patterns are uncorrelated with the prototypes. As for the SP model this model achieves a finite non-zero information capacity 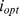 in the large 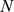 limit if the depression-potentiation ratio 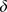 is of order one, and if the coding level scales with network size as 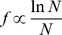. If learning is slow, 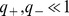, and the number of presentations of patterns of each class becomes large the probabilities 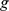 and 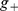 are [23]:
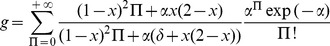
(29)and
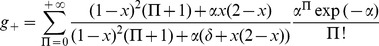
(30)


We inserted those expressions in Eqs. (19,20) to study the maximal information capacity of the network under this learning protocol. The optimal information 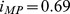 bits/synapse is reached at 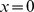 for 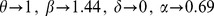 which gives 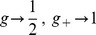. In this limit, the network becomes equivalent to the Willshaw model.

**Figure 3:**
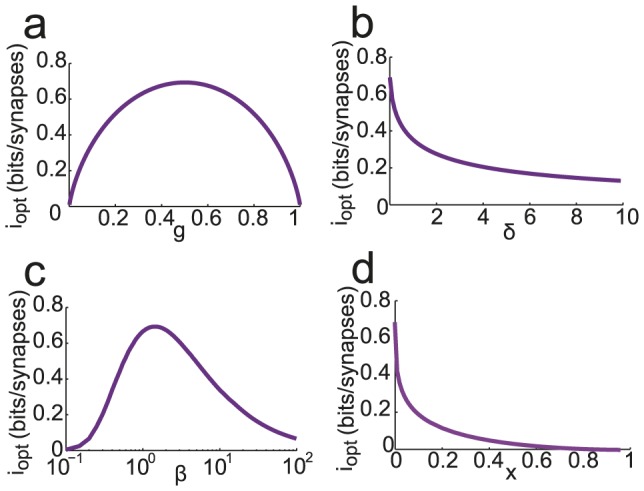
Optimized information capacity for the MP model in the limit 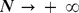. a. Optimal information capacity as a function of 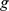, the average number of activated synapses after learning. Optimal capacity is reached in the limit 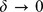 and at 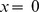 where the capacity is the same as for the Willshaw model. b. Dependence of information capacity on 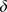, the ratio between the number of depressing events and potentiating events at pattern presentation. c. Dependence on 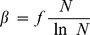. d. Dependence on the noise in the presented patterns, 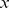. This illustrates the trade-off between the storage capacity and the generalization ability of the network.

The maximal capacity is about 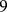 times larger than for a network that has to learn in one shot. On Figure 3a we plot the optimal capacity as a function of 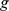. The capacity of the slow learning network with multiple presentations is bounded by the capacity of the Willshaw model for all values of 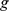, and it is reached when the depression-potentiation ratio 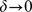. For this value, no depression occurs during learning: the network loses palimpsest properties, i.e. the ability to erase older patterns to store new ones, and it is not able to learn if the presented patterns are noisy. The optimal capacity decreases with 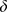, for instance at 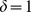 (as many potentiation events as depression events at each pattern presentation), 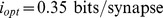. Figure 3c shows the dependence as a function of 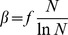. In Figure 3d, we show the optimized capacity for different values of the noise 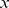 in the presented patterns. This quantifies the trade-off between the storage capacity and the generalization ability of the network [23].

### Finite-size networks

The results we have presented so far are valid for infinite size networks. Finite-size effects can be computed for the three models we have discussed so far (see Methods). The main result of this section is that the capacity of networks of realistic sizes is very far from the large N limit. We compute capacities for finite networks in the SP and MP settings, and we validate our finite size calculations by presenting the results of simulations of large networks of sizes 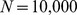, 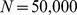.

We summarize the finite size calculations for the SP model (a more general and detailed analysis is given in Methods). In the finite network setting, conditional on the tested pattern 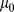 having 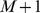 selective neurons, the probability of no error 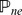 is given by
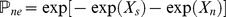
with



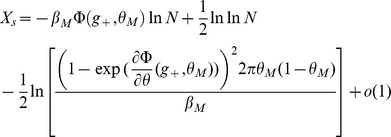


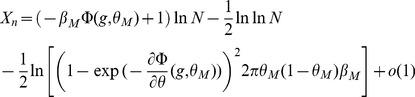
(31)


where 
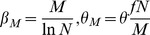
 and 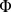 is given by Eq. (13). In the calculations for 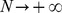 discussed in the previous sections we kept only the dominant term in 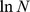, which yields Eqs. (19) and (20).

**Figure 4:**
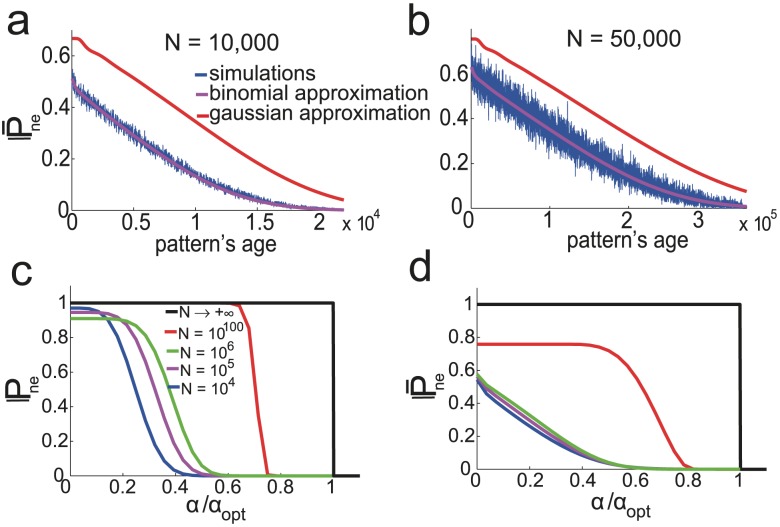
Finite size effects. Shown is 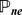, the probability that a tested pattern of a given age is stored without errors, for the SP model. a. 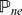 as a function of the age of the tested pattern. Parameters are those optimizing capacity at 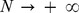, results are for simulations (blue line) and calculations with a binomial approximation of the fields distributions (magenta) and a gaussian approximation (red); 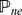 is averaged over different value of 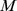, the number of selective neurons in the tested pattern (magenta line). b Same for 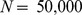. c. 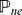 as a function of a scaled version of pattern age (see text for details), fluctuations in 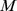 are discarded on this plot. d. Same as c with an average of 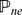 over different 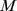.

In the above equations, the first order corrections scale as 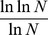, which has a dramatic effect on the storage capacity of finite networks. In Figure 4a,b, we plot 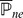 (where the bar denotes an average over the distribution of 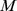) as a function of the age of the pattern, and compare this with numerical simulations. It is plotted for 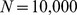 and 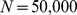 for learning and network parameters chosen to optimize the storage capacity of the infinite-size network (see Section ‘Amit-Fusi model’). We show the result for two different approximations of the field distribution: a binomial distribution (magenta), as used in the previous calculations for infinite size networks; and a gaussian (red) approximation (see Methods for calculations) as used by previous authors [19], [20], [24]. For these parameters the binomial approximation gives an accurate estimation of 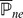, while the gaussian calculation overestimates it.

The curves we get are far from the step functions predicted for 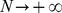 by Eq. (45). To understand why, compare Eqs. (15), and (31): finite size effects can be neglected when 
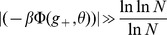
 and 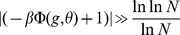. Because the finite size effects are of order 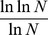, it is only for huge values of 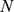 that the asymptotic capacity can be recovered. For instance if we choose an activation threshold 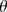 slightly above the optimal threshold given in Section ‘Amit-Fusi model’ (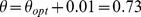), then 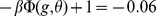, and for 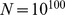 we only have 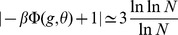. In Figure 4c we plot 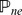 as a function of 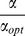 where 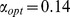 is the value of 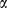 that optimizes capacity in the large 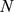 limit, 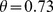 and the other parameters are the one that optimizes capacity. We see that we are still far from the large 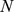 limit for 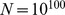. Networks of sizes 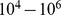 have capacities which are only between 20% and 40% of the predicted capacity in the large 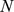 limit. Neglecting fluctuations in the number of selective neurons, we can derive an expression for the number of stored patterns 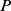 that includes the leading finite size correction for the SP model,
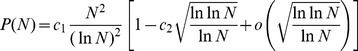
(32)where 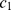 and 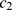 are two constants (see Methods).

If we take fluctuations in the number of selective neurons into account, it introduces other finite-size effects as can be seen from Eqs. (43) and (44) in the Methods section. These fluctuations can be discarded if 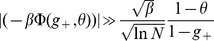 and 
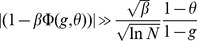
. In Figure 4d we plot 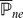 for different values of N. We see that finite size effects are even stronger in this case.

**Figure 5:**
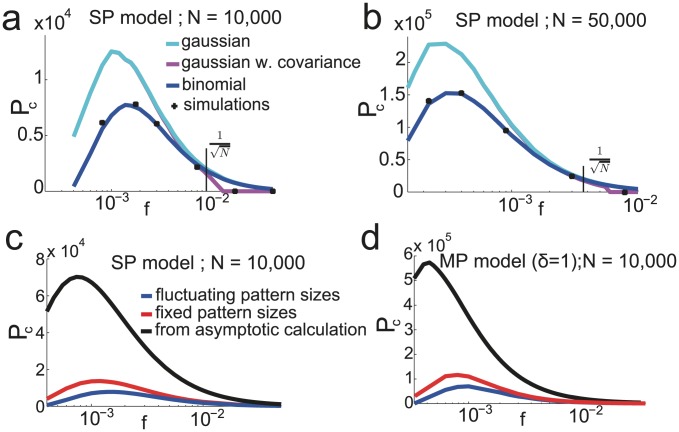
Capacity at finite 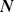. a,b. 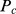 as a function of 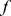 for the SP model and 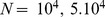 Parameters are chosen to optimize capacity under the binomial approximation. Shown are the result of the gaussian approximation without covariance (cyan) and with covariance (magenta) for these parameters. c. Optimized 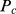 as a function of 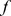 for the SP model at 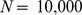. The blue curve is for patterns with fluctuations in the number of selective neurons. The red curve is for the same number of selective neurons in all patterns. The black curve is the number of patterns that would be stored if the network were storing the same amount of information as in the case 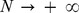. d. Same for the MP model, where parameters have been optimized, but the depression-potentiation ratio is fixed at 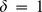.

To plot the curves of Figure 4, we chose parameters to be those that optimize storage capacity for infinite network sizes. When 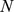 is finite, those parameters are no longer optimal. To optimize parameters at finite 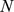, since the probability of error as a function of age is no longer a step function, it is not possible to find the last pattern stored with probability one. Instead we define the capacity 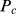 as the pattern age for which 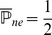. Using Eqs. (31) and performing an average over the distribution of 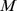, we find parameters optimizing pattern capacity for fixed values of 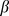. Results are shown on Figure 5a,b for 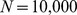 and 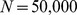. We show the results for the different approximations used to model the neural fields: the blue line is the binomial approximation, the cyan line the gaussian approximation and the magenta one is a gaussian approximation with a covariance term that takes into account correlations between synapses (see Methods and [19], [20]). For 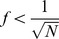 the storage capacity of simulated networks (black crosses) is well predicted by the binomial approximation while the gaussian approximations over-estimates capacity. For 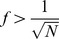, the correlations between synapses can no longer be neglected [17]. The gaussian approximation with covariance captures the drop in capacity at large 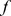.

For 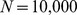, the SP model can store a maximum of 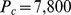 patterns at a coding level 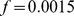 (see blue curve in figure 5c). As suggested in Figures 4c,d, the capacity of finite networks is strongly reduced compare to the capacity predicted for infinite size networks. More precisely, if the network of size 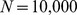 had the same information capacity as the infinite size network (27), it would store up to 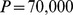 patterns at coding level 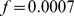. Part of this decrease in capacity is avoided if we consider patterns that have a fixed number 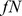 of selective neurons. This corresponds to the red curve in figure 4c. For fixed sizes the capacity is approximately twice as large. Note that finite-size effects tend to decrease as the coding level increases. In Figure 5c, 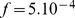, and the capacity is 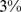 of the value predicted by the large 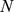 limit calculation. The ratio of actual to asymptotic capacities increases to 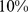 at 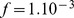 and 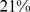 at 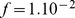. In Figure 5d, we do the same analysis for the MP model with 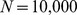. Here we have also optimized all the parameters, except for the depression-potentiation ratio which is set to 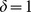, ensuring that the network has the palimpsest property and the ability to deal with noisy patterns. For 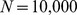, the MP model with 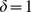 can store up to 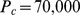 patterns, at 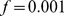 (versus 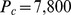 at 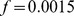 for the SP model). One can also compute the optimized capacity for a given noise level. At 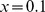, 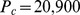 for 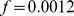 and 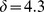 or at 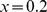, 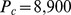 for 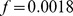 and 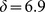.

### Storage capacity with errors

So far, we have defined the storage capacity as the number of patterns that can be perfectly retrieved. However, it is quite common for attractor neural networks to have stable fixed point attractors that are close to, but not exactly equal to, patterns that are stored in the connectivity matrix. It is difficult to estimate analytically the stability of patterns that are retrieved with errors as it requires analysis of the dynamics at multiple time steps. We therefore used numerical simulations to check whether a tested pattern is retrieved as a fixed point of the dynamics at a sufficiently low error level. To quantify the degree of error, we introduce the overlap 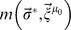 between the network fixed point 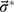 and the tested pattern 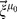, with 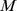 selective neurons
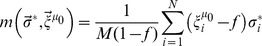
(33)


**Figure 6:**
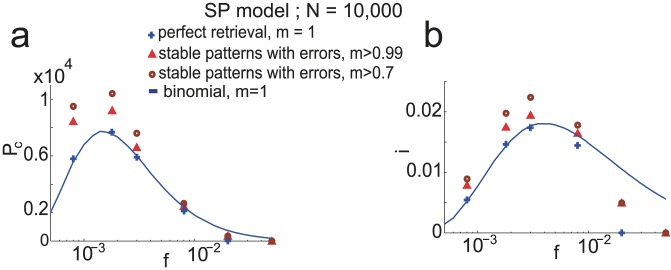
Storage capacity with errors in the SP model. Instead of counting only patterns that are perfectly retrieved, patterns that lead to fixed points of the dynamic overlapping significantly (see text for the definition of the overlap) with the tested memory pattern are also counted. Simulations are done with the same parameters as in Figure 5a. a. 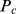 as a function of 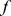. Blue crosses correspond to fixed points that are exactly the stored patterns. Red triangles correspond to fixed points that have an overlap larger than 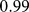, and brown circles an overlap larger than 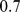. b. Same as a. but instead of quantifying storage capacity with 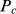, it is done with 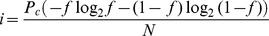.

In Figure 6a we show 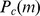, the number of fixed-point attractors that have an overlap larger than 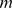 with the corresponding stored pattern, for 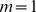, 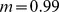 and 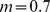. Note that only a negligible number of tested patterns lead to fixed points with 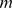 smaller than 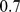, for 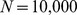 neurons. Considering fixed points with errors leads to a substantial increase in capacity, e.g. for 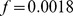 the capacity increases from 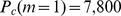 to 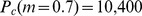. In Figure 6b, we quantify the information capacity in bits stored per synapse, defined as in Eq. (6), 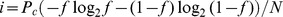. Note that in the situation when retrieval is not always perfect this expression is only an approximation of the true information content. The coding level that optimizes the information capacity in bits per synapse 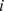 is larger (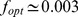) than the one that optimizes the number of stored patterns 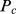 (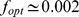), since the information content of individual patterns decreases with 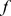. Finally, note that the information capacity is close to its optimum in a broad range of coding levels, up to 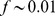.

### Increase in capacity with inhibition

As we have seen above, the fluctuations in the number of selective neurons in each pattern lead to a reduction in storage capacity in networks of finite size (e.g. Figure 5c,d). The detrimental effects of these fluctuations can be mitigated by adding a uniform inhibition 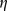 to the network [19]. Using a simple instantaneous and linear inhibitory feed-back, the local fields become
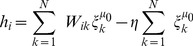
(34)


**Figure 7:**
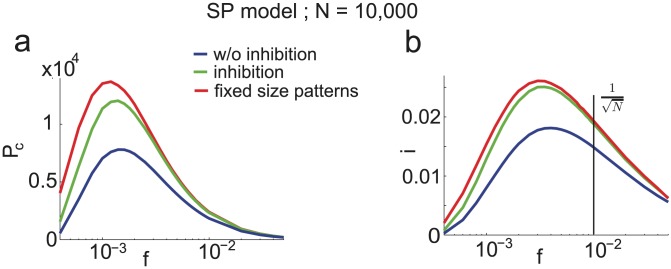
Storage capacity optimized with inhibition in the SP model. Blue is for a fixed threshold and fluctuations in the number of selective neurons per pattern. Green, the fluctuations are minimized using inhibition. Red, without fluctuations in the number of selective neurons per pattern. a. Number of stored patterns as a function of the coding level 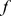. b. Stored information in bits per synapse, as a function of 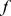.

For infinite size networks, adding inhibition does not improve storage capacity since fluctuations in the number of selective neurons vanish in the large N limit. However, for finite size networks, minimizing those fluctuations leads to substantial increase in storage capacity. When testing the stability of pattern 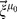, if the number of selective neurons is unknown, the variance of the field on non-selective neurons is 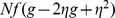, and 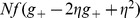 for selective neurons (for small 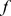). The variance for non-selective neurons is minimized if 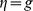, yielding the variance obtained with fixed size patterns. The same holds for selective neurons at 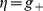. Choosing a value of 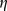 between 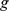 and 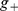 brings the network capacity towards that of fixed size patterns. In Figure 7a, we show the storage capacity as a function of 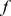 for these three scenarios. Optimizing the inhibition 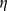 increases the maximal capacity by 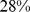 (green curve) compared to a network with no inhibition (blue curve). Red curve is the capacity without pattern size fluctuations. Inhibition increases the capacity from 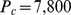 at 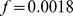 to 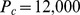. In Figure 7b, information capacity measured in bits per synapse is shown as a function of 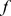 in the same three scenarios. Note again that for 
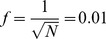
, the capacity is quite close to the optimal capacity.

## Discussion

We have presented an analytical method to compute the storage capacity of networks of binary neurons with binary synapses in the sparse coding limit. When applied to the classic Willshaw model, in the infinite limit, we find a maximal storage capacity of 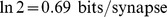, the same than found in previous studies, although with a different definition adapted to recurrent networks, as discussed in the section ‘Willshaw model’. We then used this method to study the storage capacity of a network with binary synapses and stochastic learning, in the single presentation (SP) scenario [17]. The main advantage of this model, compared to the Willshaw model, is its palimpsest property, that allows it to do on-line learning in an ever changing environment. Amit and Fusi showed that the optimal storage capacity was obtained in the sparse coding limit, 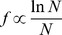 and with a balance between the effect of depression and potentiation. The storage capacity of this network has been further studied for finite size networks in [19], [20]. We have complemented this work by computing analytically the storage capacity in the large 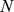 limit. The optimal capacity of the SP model is 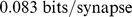, which is about 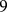 times lower than the one of the Willshaw model. This decrease in storage capacity is similar to the decrease seen in palimpsest networks with continuous synapses - for example, in the Hopfield model the capacity is about 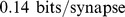, while in a palimpsest version the capacity drops to about 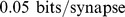. The reason for this decrease is that the most recently seen patterns have large basins of attraction, while older patterns have smaller ones. In the Willshaw model, all patterns are equivalent, and therefore they all have vanishing basins of attraction at the maximal capacity.

We have also studied the network in a multiple presentation (MP) scenario, with in which patterns presented to the network are noisy versions of a fixed set of prototypes, in the slow learning limit in which transition probabilities go to zero [23]. In the extreme case in which presented patterns are the prototypes, all synaptic weights are initially at zero, and if the synapses do not experience depression, this model is equivalent to the Willshaw model with a storage capacity of 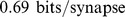, which is about 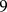 times larger than the capacity of the SP model. A more interesting scenario is when depression is present. In this case then the network has generalization properties (it can learn prototypes from noisy versions of them), as well as palimpsest properties (if patterns drawn from a new set of prototypes are presented it will eventually replace a previous set with the new one). We have quantified the trade-off between generalization and storage capacity (see Figure 3d). For instance, if the noisy patterns have 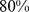 of their selective neurons in common with the prototypes to be learned, the storage capacity is decreased from 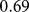 to 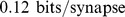.

A key step in estimating storage capacity is deriving an accurate approximation for the distribution of the inputs neurons receive. These inputs are the sum of a large number of binary variables, so the distribution is a binomial if one can neglect the correlations between these variables, induced by the learning process. Amit and Fusi [17] showed that these correlations can be neglected when 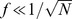. Thus, we expect the results with the binomial approximation to be exact in the large 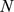 limit. We have shown that a Gaussian approximation of the binomial distribution gives inaccurate results in the sparse coding limit, because the capacity depends on the tail of the distribution, which is not well described by a Gaussian. For larger coding levels (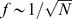), the binomial approximation breaks down because it does not take into account correlations between inputs. Following [19] and [20], we use a Gaussian approximation that includes the covariance of the inputs, and show that this approximation captures well the simulation results in this coding level range.

We computed storage capacities for two different learning scenarios. Both are unsupervised, involve a Hebbian-type plasticity rule, and allow for online learning (providing patterns are presented multiple times for the MP model). It is of interest to compare the performance of these two particular scenarios with known upper bounds on storage capacity. For networks of infinite size with binary synapses such a bound has been derived using the Gardner approach [25]. In the sparse coding limit, this bound is 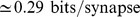 with random patterns (in which fluctuations in the number of selective neurons per pattern fluctuates), and 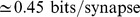 if patterns have a fixed number of selective neurons [26]. We found a capacity of 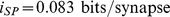 for the SP model and 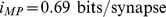 for the MP model, obtained both for patterns with fixed and variable number of selective neurons. The result for the MP model seems to violate the Gardner bound. However, as noticed by Nadal [21], one should be cautious in comparing these results: in our calculations we have required that a given pattern is stored perfectly with probability one, while the Gardner calculation requires that **all** patterns are stored perfectly with probability one. As mentioned in the section ‘Willshaw model’, the capacity of the Willshaw and MP models drops to 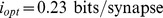 in the case of fixed-size patterns, if one insists that **all** patterns should be stored perfectly, which is now consistent with the Gardner bound. This means that the MP model is able to reach a capacity which is roughly half the Gardner bound, a rather impressive feat given the simplicity of the rule. Note that supervised learning rules can get closer to these theoretical bounds [27].

We have also studied finite-size networks, in which we defined the capacity as the number of patterns for which the probability of exact retrieval is at least 50%. We found that networks of reasonable sizes have capacities that are far from the large 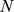 limit. For networks of sizes 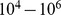 storage capacities are reduced by a factor 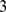 or more (see Figure 4). These huge finite size effects can be understood by the fact that the leading order corrections in the large 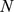 limit are in 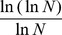 - and so can never be neglected unless 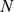 is an astronomical number (see Methods). A large part of the decrease in capacity when considering finite-size networks is due to fluctuations in the number of selective neurons from pattern to pattern. In the last section, we have used inhibition to minimize the effect of these fluctuations. For instance, for a network of 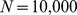 neurons learning in one shot, inhibition allows to increase capacity from 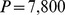 to 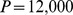. For finite size networks, memory patterns that are not perfectly retrieved can still lead to fixed points where the activity is significantly correlated with the memory patterns. We have investigated with simulations how allowing errors in the retrieved patterns modifies storage capacity. For 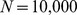, the capacity increases from 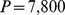 to 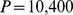, i.e. by approximately 30%.

Our study focused on networks of binary neurons, connected through binary synapses, and storing very sparse patterns. These three assumptions allowed us to compute analytically the storage capacity of the network in two learning scenarios. An important question is how far real neural networks are from such idealized assumptions. First, the issue of whether real synapses are binary, discrete but with a larger number of states, or essentially continuous, is still unresolved, with evidence in favor of each of these scenarios [28], [29], [30], [31], [32]. We expect that having synapses with a finite number 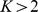 of states will not modify strongly the picture outlined here [17], [33], [20]. Second, it remains to be investigated how these results will generalize to networks of more realistic neurons. In strongly connected networks of spiking neurons operating in the balanced mode [34], [35], [36], [37], the presence of ongoing activity presents strong constraints on the viability of sparsely coded selective attractor states. This is because ‘non-selective’ neurons are no longer silent, but are rather active at low background rates, and the noise due to this background activity can easily wipe out the selective signal [35], [38]. In fact, simple scaling arguments in balanced networks suggest the optimal coding level would become 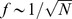 [3], [39]. The learning rules we have considered in this paper lead to a vanishing information stored per synapse with this scaling. Finding an unsupervised learning rule that achieves a finite information capacity in the large 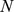 limit in networks with discrete synapses for such coding levels remains an open question. However, the results presented here show that for networks of realistic sizes, the information capacity at such coding levels is in fact not very far from the optimal one that is reached at lower coding levels (see vertical lines in Figure 5–7). Finally, the coding levels of cortical networks during delay period activity remain poorly characterized. Experiments in IT cortex [40], [41], [42] are consistent with coding levels of order 1%. Our results indicate that in networks of reasonable sizes, these coding levels are not far from the optimal values.

The SP and MP models investigated in this paper can be thought of as minimal models for learning in hippocampus and neocortex. The SP model bears some resemblance to the function of hippocampus, which is supposed to keep a memory of recent episodes that are learned in one shot, thanks to highly plastic synapses. The MP model relates to the function of neocortex, where a longer-term memory can be stored, thanks to repeated presentations of a set of prototypes that occur repeatedly in the environment, and perhaps during sleep under the supervision of the hippocampus. The idea that hippocampal and cortical networks learn on different time scales has been exploited in several modeling studies [43], [44], [45], in which the memories are first stored in the hippocampus and then gradually transferred to cortical networks. It would be interesting to extend the type of analysis presented here to coupled hippocampo-cortical networks with varying degrees of plasticity.

## Methods

### Capacity calculation for infinite size networks

We are interested at retrieving pattern 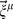 that has been presented during the learning phase. We set the network in this state 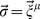 and ask whether the network remains in this state while the dynamics (2) is running. At the first iteration, each neuron 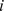 is receiving a field
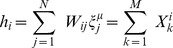
(35)


Where M+1 is the number of selective neurons in pattern 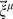, with 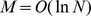. Where we use the standard ‘Landau’ notations: 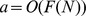 means that 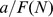 goes to a finite limit in the large 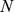 limit, while 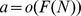 means that 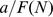 goes to zero in the large 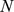 limit. and 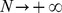. We recall that 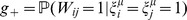 and 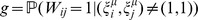. Thus 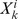 is a binary random variable which is 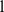 with probability, either 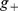 if 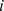 is a selective neuron (sites 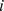 such that 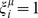), or 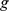 if 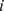 is a non-selective neuron (sites 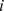 such that 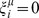). Neglecting correlations between 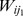 and 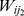 (it is legitimate in the sparse coding limit we are interested in, see [17]), the 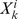
's are independent and the distribution of the field on selective neurons can be written as
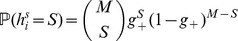


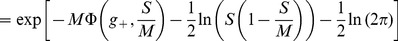
(36)where we used Stirling formula for 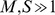, with 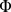 defined in (13). For non-selective neurons
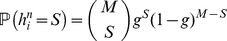


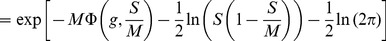
(37)


Now write
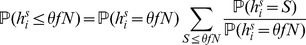


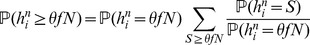
(38)


In the limit 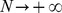 we are considering in this section, and if 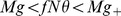, the sums corresponding to the probabilities 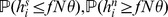 are dominated by their first term (corrections are made explicit in the following section). Keeping only higher order terms in 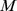 in Eqs. (36) and (37), we have:
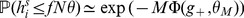
(39)and
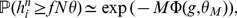
(40)


yielding Eq. (15) with 
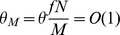
. Note that with the coding levels we are considering here (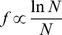), 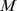 is of order 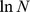. When the number of selective neurons per pattern is fixed at 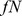, we choose 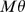 for the activation threshold and these equations become:
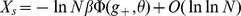


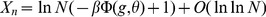
(41)where 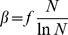


For random numbers of selective neurons we need to compute the average over 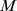: 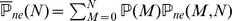. Since 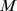 is distributed according to a binomial of average 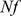 and variance 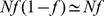, for sufficiently large 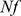, this can be approximated as 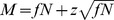 where 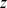 is normally distributed:
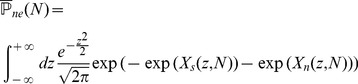
(42)with
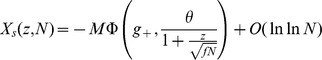


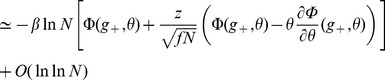


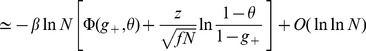
(43)and
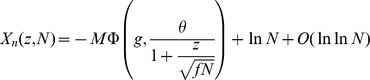


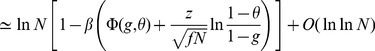
(44)


When 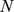 goes to infinity, we bring the limit into the integral in Eq. (42) and obtain
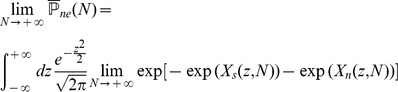


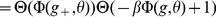
(45)where 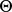 is the Heaviside function. Thus in the limit of infinite size networks, the probability of no error is a step function. The first Heaviside function implies that the only requirement to avoid errors on selective neurons is to have a scaled activation threshold 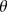 below 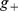. The second Heaviside function implies that, depending on 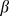, 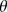 has to be chosen far enough from 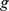. The above equation allows to derive the inequalities (19) and (20).

### Capacity calculation for finite-size networks

We now turn to a derivation of finite-size corrections for the capacity. Here we show two different calculations. In the first calculation, we derive Eq. (32), taking into account the leading-order correction term in Eq. (43). This allows us to compute the leading-order correction to the number of patterns 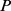 that can be stored for a given set of parameters. However, it does not predict accurately the storage capacity of the large-size but finite networks that we simulated. In the second calculation presented, we focus on computing the probability of no error in a given pattern 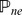, including a next-to-leading-order correction.

Eq. (32) is derived for a fixed set of parameters, assuming that the set of active neurons have a fixed size, and that the activation threshold 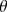 has been chosen large enough such that the probability to have non-selective neurons activated is small. From the Stirling expansion, adding the first finite-size correction term in Eq. (41), we get
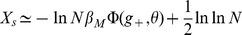
(46)with 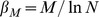. For large 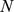, the number of stored patterns 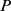 can be increased until 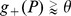. Setting 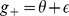, an expansion of 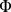 in 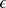 allows to write
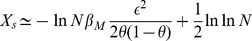
(47)


The 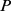 patterns are correctly stored as long as 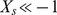. This condition is satisfied for 
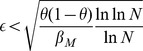
. For the SP model, we can deduce which value of 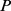 yields this value of 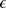 (see Eq. (26)). This allows to derive Eq. (32),
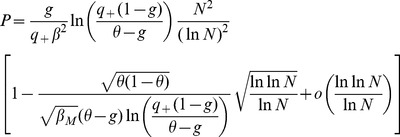
(48)


We now turn to a calculation of the probability of no error on a given pattern 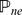, taking into account the next-to-leading order correction of order one, in addition to the term of order 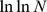 in Eq. (41). This is necessary to predict accurately the capacity of realistic size networks (for instance for 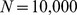, 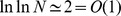). 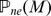 is computed for a memory pattern with 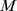 selective neurons. The estimation of 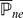 used in the figures is obtained by averaging over different values of 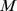, with 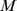 drawn from a binomial distribution of mean 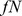.

We first provide a more detailed expansion of the sums in Eq. (38). Setting 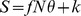, with the Taylor expansions:
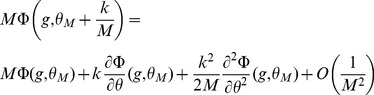
(49)

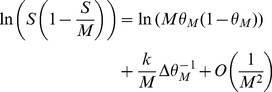
(50)where 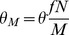 and 
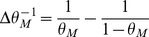
. Using (37) we can rewrite:
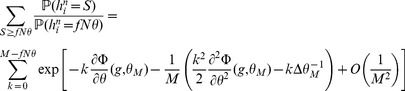
(51)


In the cases we consider, we will always have 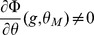 so that we can consider only the term of order 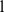 in 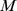. The sum is now geometric, and we obtain
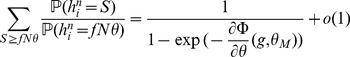
(52)


The same kind of expansion can be applied for the selective neurons. Again if we are in a situation where 
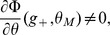


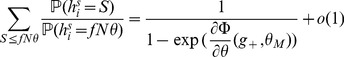
(53)


When 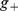 is close to 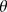 and thus 
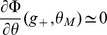
, we are then left with:
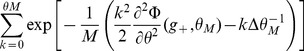
(54)

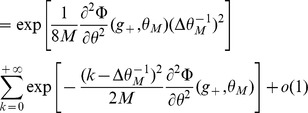


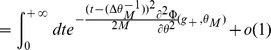


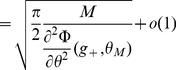
(55)


When 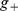 is too close to 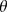, which is the case for the optimal parameters in the large 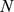 limit, we need to use (55). It only contributes a term of order 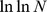 in 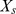 and does not modify our results. In Figures 6-7, we use (53), which gives from (38) and (36), (37) and (53),(52):
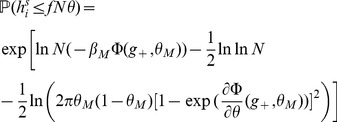
(56)

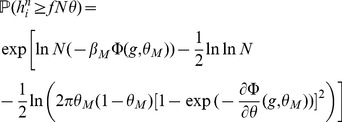
(57)


The probability of no error is
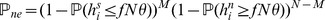


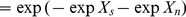
(58)which leads to Eqs. (31)
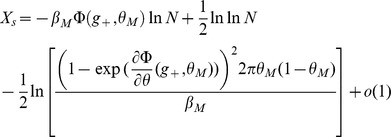


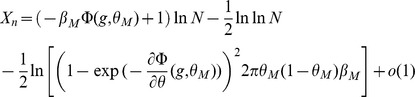



### Gaussian approximation of the fields distribution

For a fixed number 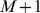 of selective neurons in pattern 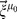, approximating the distribution of the fields on background neurons 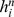 and selective neurons 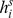 with a gaussian distribution gives:
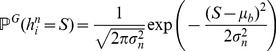
(59)where
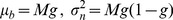
(60)and
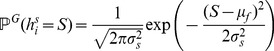
(61)where
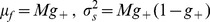
(62)


The probability that those fields are on the wrong side of the threshold are:
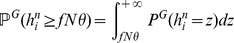
(63)and
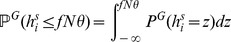
(64)


Following the same calculations presented, and keeping only terms that are relevant in the limit 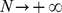, the probability that there is no error is given by:
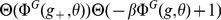
(65)where the rate function 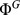 is
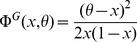
(66)


Calculations with the binomial versus the gaussian approximation differ only in the form of 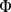. Finite size terms can be taken into account in the same way it is done in the previous Methods section for the binomial approximation.

In all above calculations we assumed that fields are sums of independent random variables (35). For small 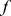 correlations are negligible [17], [19]. It is possible to compute the covariances between the terms of the sum (see Eq. (3.9) in [19]), and take them into account in the gaussian approximation. This can be done using
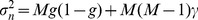
(67)

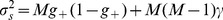
(68)in Eqs. (59),(61), where
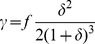
(69)

